# Assessment of the Quality and Reliability of Content Available on YouTube About Palpitations

**DOI:** 10.7759/cureus.58710

**Published:** 2024-04-22

**Authors:** Laxmi Priya Seelam, Rohan S Chippada, Kshitij Raj, Shrishti Agarwal, Fetsum Tekalegn, Akash Santhosh, Aakriti Tiwari

**Affiliations:** 1 Internal Medicine, MNR Medical College and Hospital, Sangareddy, IND; 2 Internal Medicine, Jawaharlal Institute of Postgraduate Medical Education and Research, Puducherry, IND; 3 Internal Medicine, Gokuldas Tejpal Hospital, Mumbai, IND; 4 College of Medicine, BJ Medical College, Ahmedabad, IND; 5 Internal Medicine, Addis Ababa University (AAU) Medical Faculty, Addis Ababa, ETH; 6 Internal Medicine, Kasturba Medical College, Mangalore, IND; 7 Internal Medicine, KJ Somaiya Medical College, Mumbai, IND

**Keywords:** fast heartbeat, global quality score, observational study, cross sectional study, youtube, palpitations

## Abstract

Palpitations refer to the sensation of rapid, fluttering, or pounding heartbeats in the chest, the determinants of which may range from hormonal changes to anxiety or arrhythmias. YouTube is one of the most prevailing and accepted web-based platforms people trust to help them understand more about their health conditions. Thus, this study aims to assess whether the quality of content about palpitations on this platform is reliable and sufficient. Seventy-one YouTube videos were analyzed using criteria such as date and time of upload, type of uploader, and type of content. The Global Quality Score (GQS) and modified DISCERN score were used to analyze the quality and reliability of the information provided. Microsoft Excel (Microsoft Corporation, Redmond, WA, US) was used for data analysis, and StataCorp’s 2023 Stata Statistical Software (College Station, TX, US) was used for statistical analysis and visualization. Of the 71 videos analyzed, 90.14% were uploaded more than a year ago, 80.28% described the symptomatology in detail, and 81.69% accurately described the etiological factors. Hospitals and doctors were the most common uploaders, constituting 23% and 19% of the uploaded videos, respectively, and had high GQSs (Median GQS = 4). The highest scores also belonged to videos uploaded by patients suffering from the disease (Median GQS = 5). Hospitals and news channels ranked highest on the reliability score (Median DISCERN = 4, respectively). It was determined that despite varied sources, the nature of content provided by the platform contains promotional material and content gaps; YouTube should, therefore, be used critically and as per professional sources.

## Introduction

A palpitation is an uncomfortable feeling in the chest described by a racing, fluttering, or pounding sensation or a sense of the heart having skipped a beat. These sensations are mostly considered normal clinically unless accompanied by overt signs and other associated symptoms such as syncope, chest pain, and a feeling of doom [1]. These sensations are sometimes felt along the neck and shoulders. Palpitations are experienced by persons of all genders, occupations, and classes and are one of the few conditions not divided by geographical location [2]. Palpitations are caused by various factors, namely, stress, anxiety, exercise, drugs, valvular heart diseases, ischemic heart diseases, rhythm disorders, and other electrophysiological conditions of the heart.

In the twenty-first century, information is widely available and easily accessible for almost any topic. Information can be obtained from the internet via a long list of apps, one of which is YouTube. YouTube is an American online video-sharing and social media platform launched on February 14, 2005. As a modality for providing medical information and education, YouTube is a relatively young idea that has bloomed into a fairly common method in technologically advanced countries [3]. Most creators include medical students, teachers, doctors, specialists, private clinics, and hospital chains, and they provide viewers with information about disease statistics, symptoms, signs, and various treatment plans [4-6]. An interesting aspect is patients and families posting content about suffering from the disease, navigating its course, and dealing with the pain and emotional burden [7]. These posts have given viewers relief and upliftment and helped decrease the social stigma of major diseases.

Currently, there are two billion active YouTube users in the world. Most users use YouTube to get information about diseases affecting themselves and their loved ones. So, providing clinical, statistical, and most recent information is necessary when dealing with social media platforms serving a huge chunk of the world's population [8]. Palpitations as content on YouTube require expertise since many people can commonly encounter them [9]. However, discussion about dangerous palpitations and their treatment should be emphasized because it may help with a patient's quality of life, morbidity, and survival.

This study aims to evaluate the quality and reliability of information provided on YouTube videos regarding palpitations and to analyze the content as a reliable source of health information. It also aims to study the popularity of YouTube videos using metrics like views, likes, and comments. Further, the paper aims to check the accuracy and trustworthiness of online resources for health information on palpitations and examine the identity and expertise of YouTube video uploaders.

## Materials and methods

This cross-sectional, observational study was conducted on a single day on November 20, 2023, as the number of likes, views, and comments on YouTube videos keeps changing. The study was exempt from Institutional Ethics Committee approval, as no human participation was involved.

A specific questionnaire with predetermined criteria was prepared using Google Forms. First, we assessed the time since these videos were uploaded, by whom they were uploaded, and the number of views, likes, and comments they received. Then, we determined the type of information provided by the video, looking for descriptions of etiology, investigations, prevention, treatments, rehabilitation, mortality, support groups, patient experience, and whether or not companies or doctors presented any promotional content. Third, we assessed the quality and reliability of the content they shared using the Global Quality Score (GQS) and the DISCERN tool, respectively [10,11].

Each of the seven authors was assigned one keyword, such as "palpitation, "fast heart rate," "palpitation prevention," "palpitation treatment," "fast heartbeat," "palpitation reason," and "fast heartbeat treatment." Each author analyzed and evaluated 15 videos for the keywords assigned. The inclusion criteria were videos relevant to the topic, video length between one and 20 minutes, and English or Hindi language. The responses were recorded in Google Sheets (Google LLC, Menlo Park, California, US) and transferred to Microsoft Excel (Microsoft Corporation, Redmond, WA, US), after which the Kruskal-Wallis test was employed to compare the median scores of different groups, using StataCorp's Stata Statistical Software, 2023 (College Station, TX, US).

## Results

A total of 105 YouTube videos were evaluated. After applying inclusion and exclusion criteria and deleting repeats, 71 were finally included in this study. The cumulative views for all the videos were 14,532,527, the likes were 213,832, and the comments were 21,189.

Table 1 shows the characteristics of the YouTube videos analyzed. Overall, 90% of the videos were uploaded more than a year ago. Hospital-run accounts uploaded 32% while doctor-run accounts accounted for nearly 27%.

**Table 1:** Characteristics of the YouTube videos analyzed

Characteristic	N (%)
Time since uploaded	
More than a week to last one year (<365 days)	7 (9.85)
More than one year (>365 days)	64 (90.14)
Type of uploader	
Doctor	19 (26.76)
Hospital	23 (32.39)
Healthcare organization	5 (7.04)
Patients suffering from disease	3 (4.23)
News channels	5 (7.04)
Other	16 (22.54)

Table 2 describes the information on palpitations in the YouTube videos. Overall, 80% of the videos described the symptoms of palpitations while 81% provided information about their causes.

**Table 2:** Information on palpitations in YouTube Videos

Variable	N (%)
Description of symptoms	57 (80.28)
Information about cause/etiology?	58 (81.69)
Information about investigations/tests	25 (35.21)
Information about prevention/vaccines	32 (45.07)
Information about treatment	42 (59.15)
Information about mortality	5 (7.04)
Information about rehabilitation	9 (12.68)
Information about support groups	3 (4.23)
Information about people/patients sharing their own experience	17 (23.94)
Information about parent sharing their experience with their family members	4 (5.63)
Does the post have promotional content by pharmaceutical companies or by doctors?	8 (11.27)

Table 3 compares the GQS and reliability scores based on the uploader type, i.e. doctors, hospitals, healthcare organizations, news channels, patients suffering from the disease, and others. Patients suffering from the disease had the highest median GQS score (5), but the lowest median reliability score (2). Hospitals and news channels were observed to have the highest median reliability score (4). The p values in the Kruskal-Wallis test exceeded 0.05, and thus, it was concluded that there was no statistically significant difference in the scores depending on the uploader type.

**Table 3:** Comparison of GQS, reliability score based on the type of uploader Footnote: Values are mentioned as median (IQ1, IQ3) where IQ stands for interquartile range. p value <0.05 is statistically significant.

Statistical Test	Type of Uploader						
	Doctors (n=19)	Hospital (n=23)	Healthcare organization (n=5)	Other (n=16)	News channel (n=5)	Patients suffering from the disease (n=16)	Kruskal-Wallis test
GQS - Median (IQ1, IQ3)	4 (3,4)	4 (3,4)	4 (3,4)	4 (3,5)	5 (4,5)	5 (2,5)	0.738
Reliability Score - Median (IQ1, IQ3)	3 (3,4)	4 (2,4)	3 (3,4)	3.5 (3,4.5)	4 (3,5)	2 (2,4)	0.662

## Discussion

The internet's easy accessibility has made social media a powerful communication tool in today's world. Many viewers rely on platforms such as YouTube to acquire information regarding various aspects of life, the most important being healthcare.

Our study aimed to analyze the quality and reliability of content about palpitations that is available on YouTube. Not many similar studies are available. Palpitations are a common symptom that may be indicative of anxiety, panic attacks, an underlying cardiac problem, or a metabolic disorder. Further, a significant percentage of patients with palpitations have no known psychological or cardiovascular causes [12].

We included 71 videos, of which 64% were uploaded over a year ago and were not updated. Suresh et al. also had a similar finding, wherein around 71.2% of the videos they analyzed were uploaded more than a year ago [13].

Most of the videos (42%) in the present study were uploaded by hospitals and doctors. A similar finding was noted by Salah et al., Askin et al., and Unal-Ulutatar et al., where most of the videos were uploaded by doctors [14-16].

In our study, the cumulative number of views on these videos was 14,532,527, similar to the finding in the study conducted by Kunze et al., who analyzed 50 videos and reported 14,141,285 views [17]. The large number of views indicates people's reliance on YouTube as a source of information for medical conditions such as palpitations.

Of the videos analyzed, most were about etiology (81.69%) and symptoms (80.28%), followed by investigations (35.21%). Similar findings were observed by Bhoot et al., who found that 35.59% of videos had information regarding investigations [18]. In our study, 45.07% of videos had information about prevention and vaccines. Clarke et al. and Nanda et al. reported similar findings [19,20].

Our study found that 59.15% of the videos were regarding the treatment of palpitations. Similarly, Hogle et al. reported that 52.94% of the videos they studied had treatment information [21]. Fewer videos we studied had content regarding support groups (4.23%). Clarke et al. reported that only 3.75% of the videos they studied had information about support groups while Nanda et al. reported that 5.48% contained information on support groups [19,20]. In our study, videos sharing information about rehabilitation were 12.68%, and videos uploaded by people sharing their own experience or that of family members were 29.57%. Nanda et al. also had similar findings [20]. Our study found few videos regarding the mortality (7.04%) associated with palpitations. Notably, only 11.27% of videos had promotional content by pharmaceutical companies or doctors. Hogle et al. reported similar results (15.69%) [21].

The present study found no significant association between quality and reliability scores with the uploader type, which was also observed by Kunze et al. [16]. Overall, median GQS scores based on uploader type were found to be of good quality, with news channels and patients suffering from the disease having the highest median GQS scores (5, respectively). Holge et al. also found that the YouTube videos they examined regarding myocardial infarction exhibited high-quality content, supported by a higher average GQS score [21]. These findings contrast those by Toprak et al., Osman et al., and Zhang et al., who observed poor quality of the analyzed videos in their respective studies [22-24]. Uzun et al. further noted that YouTube videos could not be considered accurate and reliable sources [25].

This study also found reliability of videos was not significantly associated with uploader type, and found low median reliability scores among the various uploader types, as well. In contrast, Uz et al. found that YouTube videos on spasticity were reliable and high-quality [26].

Limitations

The main limitation of the present study is the inclusion of a limited number of videos. Furthermore, likes and views can change daily, and there is a possibility of new content uploaded, with better quality and reliability, which might have been missed. There is also a possibility of inter-observer bias.

## Conclusions

This study analyzed 71 YouTube videos on 'palpitations' obtained using various keywords. We found diverse contributors, including hospitals, doctors, and healthcare organizations. While the content was comprehensive regarding symptoms and etiology, gaps in information about support groups and rehabilitation were identified. Despite examining varied sources, information quality did not significantly differ across uploaders. However, the presence of promotional content highlights commercial interests. Examining the Global Quality Scale and reliability scores revealed a similar level of information quality across the analyzed YouTube videos.

In conclusion, YouTube is a valuable resource for information on palpitations. However, it is essential to address content gaps and ensure objectivity. Collaborative efforts among content creators, healthcare professionals, and platform administrators are crucial for a reliable health information environment. Users must approach online health content critically and advocate for responsible dissemination.
